# The effect of duration between sessions on microperimetric biofeedback training in patients with maculopathies

**DOI:** 10.1038/s41598-024-63327-x

**Published:** 2024-05-31

**Authors:** Jie Zhou, Jintong Hou, Shengnan Li, Jinglin Zhang

**Affiliations:** 1 Aier School of Ophthalmology, Central South University, Changsha, 410000 Hunan China; 2 Department of Epidemiology and Biostatistics, Dornsife School of Public Health, Drexel University, Philadelphia, PA 19104 USA; 3 Sichuan Eye Hospital, Aier Eye Hospital Group, Chengdu, 610047 Sichuan China; 4 Guangzhou Aier Eye Hospital, Jinan University, Guangzhou, 510040 Guangdong China

**Keywords:** Macular degeneration, Retinal diseases

## Abstract

Aim of this study was to explore the optimal training interval and times of microperimetric biofeedback training (MBFT) in maculopathies. Twenty-nine patients with maculopathies were divided into two groups: daily training (Group A) or alternate daily training (Group B). Both groups underwent 15 MBFT sessions. We compared the BCVA, reading speed, and fixation stability at baseline, after 5, 10, 15 sessions. After 15 sessions of MBFT, all visual parameters in both groups improved. There was a significant increase in BCVA after 5 sessions in both groups (P=0.016, and P<0.001 respectively), but Group A showed further improvement after 10 sessions (P<0.001). Regarding reading speed, Group A showed significant improvement from baseline after 15 sessions(P=0.020), Group B improved significantly after 5 sessions (P=0.047) and continued to improve after 10 sessions (P=0.030). Additionally, P1 and LgBCEA of Group A significantly improved after 10 sessions (P=0.001, and P=0.001 respectively), while Group B significantly improved after 5 sessions (P=0.002, and P<0.001 respectively). There was no significant difference in visual outcomes between the two groups (P>0.05) except LgBCEA (P=0.046) after 15 sessions. We concluded that the both MBFT frequencies are effective at improving vision and quality of life in patients with maculopathies. The alternate daily training group showed less time-dependent of improvement in all parameters and a greater benefit in fixation stability. Ten sessions are the optimal number of treatment sessions for alternate daily training.

## Introduction

The macular is the center of the retina and comprises a very high density of cone photoreceptors, which are responsible for almost all photopic vision. There are approximately 225,000 cones within two millimeters of the macular fovea, accounting for 25% of all ganglion cells in the brain. However, the foveal region is vulnerable to degeneration due to changes in blood flow, oxygen and nutrient delivery caused by many macular diseases^1^. Macular diseases, such as age-related macular degeneration (AMD), myopic macular degeneration (MMD), and Stargardt's disease (STGD), are characterized by impairments of the photoreceptors in the macula, which in turn lead to disorders in daily work tasks, such as driving, reading, and facial recognition, seriously affecting the quality of life of patients^2^. MMD and AMD have been the leading causes of irreversible low vision and blindness in recent years due to an aging population and increasing prevalence of myopia^3,4^.

General treatments like medicine or surgery do not provide obvious benefits to patients with macular damage because of atrophy of the choroid and degeneration of retinal pigment epithelium (RPE) and photoreceptor cells^5–7^. However, the brain is able to work around this. To compensate for the lack of central fixation, the brain automatically chooses an area of the retina outside the central macular to form eccentric fixation, known as the preferred retinal locus (PRL)^2,8^. This common adaptive strategy is a sign of neural plasticity, consistent with the hypothesis of partial cortical reorganization^9^. However, the proceed is lengthy and unpredictable, as such spontaneously-generated PRL are not always optimum^10^.The ideal PRL is the retinal locus closest to fovea with highest retinal sensitivity, offering best potential visual acuity^11^. Microperimetry biofeedback training (MBFT) has proven to be a promising efficient modality for the development of a newly trained retinal locus (TRL) or for strengthening the spontaneous PRL to achieve better visual performance.

The rationale of MBFT therapy is to reeducate the visual system to adapt to a new visual condition by promoting the transmission of the retina and brain and enhancing synaptic plasticity and neural capacity by acoustic biofeedback or structured light stimulus biofeedback^12^. Many studies have investigated improvements in patients’ visual acuity (VA), reading speed, fixation stability, and quality of life after MBFT^13–17^. However, the design and scheme of these different studies may have differed, and the conclusions may not be the same. Currently, there is no standardized criterion for MBFT, and no study has systematically compared the effects of different programs on training outcomes^18^. It is therefore extremely important to determine an appropriate program consisting of frequency, intensity, and duration of specified treatments while creating training plans for recipients of visual rehabilitation services.

This study aimed to investigate the effects of training frequency and number of training sessions on the visual outcomes of patients with various macular diseases. Fifteen training sessions were conducted at two distinct frequencies; once a day and once every other day. Baseline measurements including fixation stability, reading speed, and best-corrected visual acuity (BCVA), were obtained and followed up after 5, 10, and 15 training sessions. By comparing the changes in these visual function parameters across different stages of training, this study aimed to identify and analyze the underlying patterns and rules governing the training process. Ultimately, the results of this study could serve as a valuable reference for standardizing the use of the MBFT in clinical practice.

## Materials and methods

A prospective, comparative, non-randomized study was conducted at the Guangzhou Aier Eye Hospital. This study was approved by the institutional review board of Guangzhou Aier Hospital (GZAIER2018IRB11), and adhered to the tenets of the Declaration of Helsinki. Informed consent was obtained from all patients in this study. The trial was registered (15/06/2023) under the ClinicalTrials.gov Identifier: NCT05904444.

The inclusion criteria for the study were as follows: (1) patients diagnosed with macular disease with a BCVA worse than 20/60, (2) a stable fundus lesion on fundus examinations, (3) an education level beyond third grade, (4) no other effective treatment, and (5) were willing to improve visual quality. Exclusion criteria included: (1) ocular treatments in the preceding 3 months; (2) active fundus lesions such as inflammation, bleeding, exudation, and edema; (3) obvious opacity of the refractive media, such as keratopathy, severe cataract, or severe vitreous opacity; and (4) inability to attend scheduled follow-up appointments. If both eyes met the inclusion criteria, they were included in the same group.

### Examinations

All patients selected underwent a comprehensive ophthalmological examination at baseline, including BCVA, intraocular pressure (IOP), slit-lamp, dilated fundus photography, Optical Coherence Tomography (OCT), microperimetry examination, reading speed, and 25-item National Eye Institute Visual Function Questionnaire (NEI VFQ-25). BCVA was assessed with the Early Treatment Diabetic Retinopathy Study (ETDRS) chart. Reading speed was evaluated using the Chinese version of the International Reading Speed texts (IReST)^19^. The NEI VFQ-25 was further used to determine the influence of eye symptoms and visual impairment on daily life, which are attributed to the limitation of patients' social function and activities^20^. The questionnaires were administered by the same investigator. For patients with poor near acuity, the investigator asked the patients orally and recorded their answers. Otherwise, patients completed the questionnaires themselves.

The microperimetry examination was using a Macular Integrity Assessment device (MAIA; CenterVue, Padova, Italy). MAIA microperimetry uses a sensitive scanning laser ophthalmoscopy (SLO) and eye-tracking system to precisely correspond the macular retinal structures with visual functions. The working distance of MAIA was 30 cm, the background luminance was 4 asb, the fixation target was a red ring vision marker, the stimulation marker was Goldmann III, the stimulation luminance was 0.25asb-1000asb, the duration of the marker was 200 ms, and the dynamic stimulation range was 0–36 dB^21^. The instrument automatically calculates and displays accurate data regarding macular sensitivity, fixation stability, location and size of the scotoma, and position of the PRL. There are two ways to measure fixation stability: (1) measurement of the percentage of fixation points within the circle of one degree and two degrees, representing P1 and P2 respectively or (2) calculating the 63% or 95% bivariate contour ellipse area (BCEA), which represents 63% or 95% of the area of an ellipse that encompasses a given proportion of fixation points based on standard deviations of the horizontal and vertical eye positions during the fixation procedure^21^. Of the four indexes, P1 and 95% BCEA are the most representative, and higher P1 and lower 95% BCEA values indicate a better fixation capacity^22^.

### Procedure

The MBFT training process comprised two steps:

- (1) Choice of the Fixation Training Target(FTT): The selection of FTT was performed based on the microperimetry result and the following criteria: (1) PRL as FTT if the natural PRL is within an area of good retinal sensitivity and in a convenient location or (2) set an FTT with good retinal sensitivity, closest to the fovea, and choose the upper side of the natural PRL as far as possible, which presents the inferior visual field; this criterion is based on our extensive literature review and clinical experience spanning several years^23,24^.
- (2) Performance of MBFT: The built-in MBFT module of the MAIA microperimetry was utilized for training. During training, patients were instructed to rotate their eyeballs to find the 1-degree area of the FTT and to maintain this position as long as possible. Once they fixed the fixation target, the sound of the machine turned into a continuous beep with a white light on, and this feedback helped the patients maintain a high level of attention. Experienced trainers supervised the training process, which was conducted over 15 sessions, each lasting 10 min. The training sessions were conducted either every day or every other day, based on the patients’ time available to commute to the hospital and receive training.

### Follow up

One hour after the 5th, 10th, and 15th training sessions, the patients underwent follow-up examination for BCVA, reading speed, and microperimetry using the follow-up mode. After the final training session, the patients completed the NEI-VFQ25 questionnaire again to evaluate whether the training had improved their visual quality of life.

### Statistical Analysis

Statistical Package for the Social Sciences version 25.0 (SPSS, Inc., Chicago, IL, USA) Windows software package was used for the analyses. The Shapiro–Wilk test was used to test the normality of the quantitative variables. The BCEA values were log-transformed (LgBCEA) before further analysis as they were not normally distributed. The paired-samples t-test was used to compare statistical differences in continuous variables before and after biofeedback, and repeated-measurement analysis of variance was used to compare changes during the training process. Categorical variables were tested using Fisher’s exact test. The Mann–Whitney U test was used to compare the differences between the two groups. Statistical significance was set at p < 0.05.

## Results

### Demographic data and clinical characteristics

A total of 29 patients (35 eyes) with various macular diseases were included in the study; 16 patients (20 eyes) were enrolled in Group A (daily training) and 13 patients (15 eyes) were enrolled in Group B (training on alternate days). Of the 29 patients, seven had AMD, six had MMD, six had undergone retinal detachment surgery, six had STGD, four had undergone macular hole surgery, and two had cone degeneration. As shown in Table 1, there were no statistically significant differences in age, sex, eye, educational level, BCVA, or reading speed between the two groups (Supplementary information).

**Table 1:** Patient characteristics for both groups in baseline.

	Group A (n = 16)	Group B (n = 13)	P-value
Age, years (Mean ± SD)	55.43 ± 21.14	43.46 ± 20.90	0.139†
Female sex (n, %)	6(37.5%)	5(38.5%)	> 0.999§
Left eye (n, total number of eyes trained)	9/20	7/15	> 0.999§
Education level years (Mean ± SD)	11.94 ± 4.06	12.15 ± 4.08	0.888†
Mean BCVA (Mean ± SD), (ETDRS letters)	31.15 ± 11.35	36.40 ± 14.01	0.229†
Mean reading speed (Mean ± SD), (words/min)	70.40 ± 49.59	78.69 ± 57.43	0.734†

Group A, daily training; Group B, training on alternate days.

*BCVA* best corrected vision acuity, reading speed, reading speed, *SD* standard deviation.

A value of P < 0.05 was considered as significant.

§fisher's exact test.

†Mann–Whitney U test.

### Outcome measures at baseline and after 15 sessions

Both groups showed significant improvements in BCVA, reading speed, fixation stability and NEI-VFQ score after 15 sessions. In Group A, the initial mean BCVA of 31.15 ± 11.35 letters improved to a mean of 34.55 ± 12.32 letters (P < 0.001) after the final training session. Reading speed also significantly increased from an average of 70.40 ± 49.59 words/min to 84.20 ± 66.21 words/min (P = 0.003). Additionally, there was a significant improvement in NEI-VFQ score, from a mean of 80.82 ± 10.66 to 82.41 ± 8.61 (P = 0.031). In group B, the initial mean BCVA of 36.40 ± 14.01 letters improved to 40.47 ± 14.86 letters (P < 0.001), while reading speed significantly increased from an average of 78.60 ± 57.43 words/min to 95.33 ± 69.57 words/min (P = 0.003). A significant difference was also identified for NEI-VFQ score, which improved from a mean of 84.50 ± 4.50 to 86.25 ± 4.65 (P = 0.031). However, there was no statistically significant difference between the two groups in the change in visual level (P > 0.05) except for LgBCEA(P = 0.046) (Table 2). There was no significant difference in P1 and LgBCEA between the better eye (BE) and the worse eye (WE) in patients who underwent MBFT in both eyes (P = 0.107, and P = 0.796, respectively) (Table 3).

**Table 2:** Outcomes of BCVA, reading speed, Quality of life between two group after 15 sessions biofeedback training.

Visual outcome	Group A			Group B			Group A vs Group B		
	Pre	Post	P	Pre	Post	P	A	B	P
BCVA	31.15 ± 11.35	34.55 ± 12.32	< 0.001	36.40 ± 14.01	40.47 ± 14.86	< 0.001	3.40 ± 2.14	4.07 ± 3.08	0.610†
Reading speed	70.40 ± 49.59	84.20 ± 66.21	0.003	78.60 ± 57.43	95.33 ± 69.57	< 0.001	9.00(19.75)	14.00(27.00)	0.298†
NEI-VFQ 25 score	80.82 ± 10.66	82.41 ± 8.61	0.031	84.50 ± 4.50	86.25 ± 4.65	0.001	1.59 ± 2.76	1.75 ± 1.29	0.455†
P1	34.60 ± 17.25	45.70 ± 15.81	0.001	60.60 ± 28.64	76.67 ± 21.54	0.002	11.10 ± 13.27	16.07 ± 16.14	0.325†
LgBCEA	1.39 ± 0.23	1.21 ± 0.22	0.001	1.00 ± 0.60	0.63 ± 0.52	< 0.001	–0.18 ± 0.20	–0.36 ± 0.32	0.046†*

† Mann–Whitney U test.

*P-value statistically significant.

**Table 3:** Comparison of WE with BE.

	P1(%)	95%BCEA (deg2)
WE	20.00 ± 20.37	− 12.83 ± 15.78
BE	9.67 ± 10.46	− 12.22 ± 13.93
t	− 1.965	− 0.272
P	0.107	0.796

WE (worse eye). BE (Better eye).

### Changes over training times

**Figure 1:**
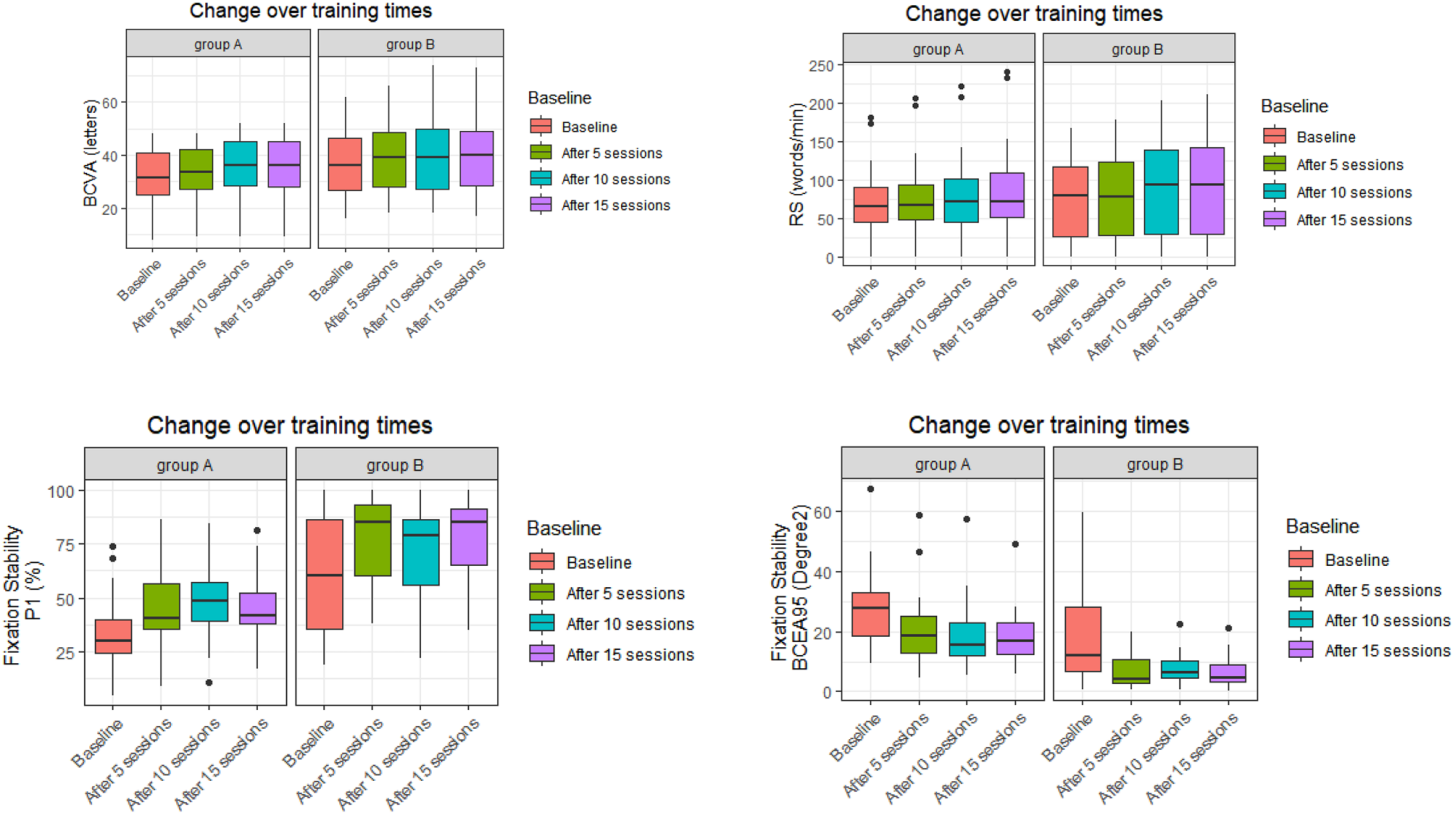
Changes in BCVA, reading speed and fixation stability over training sessions.

**Figure 2:**
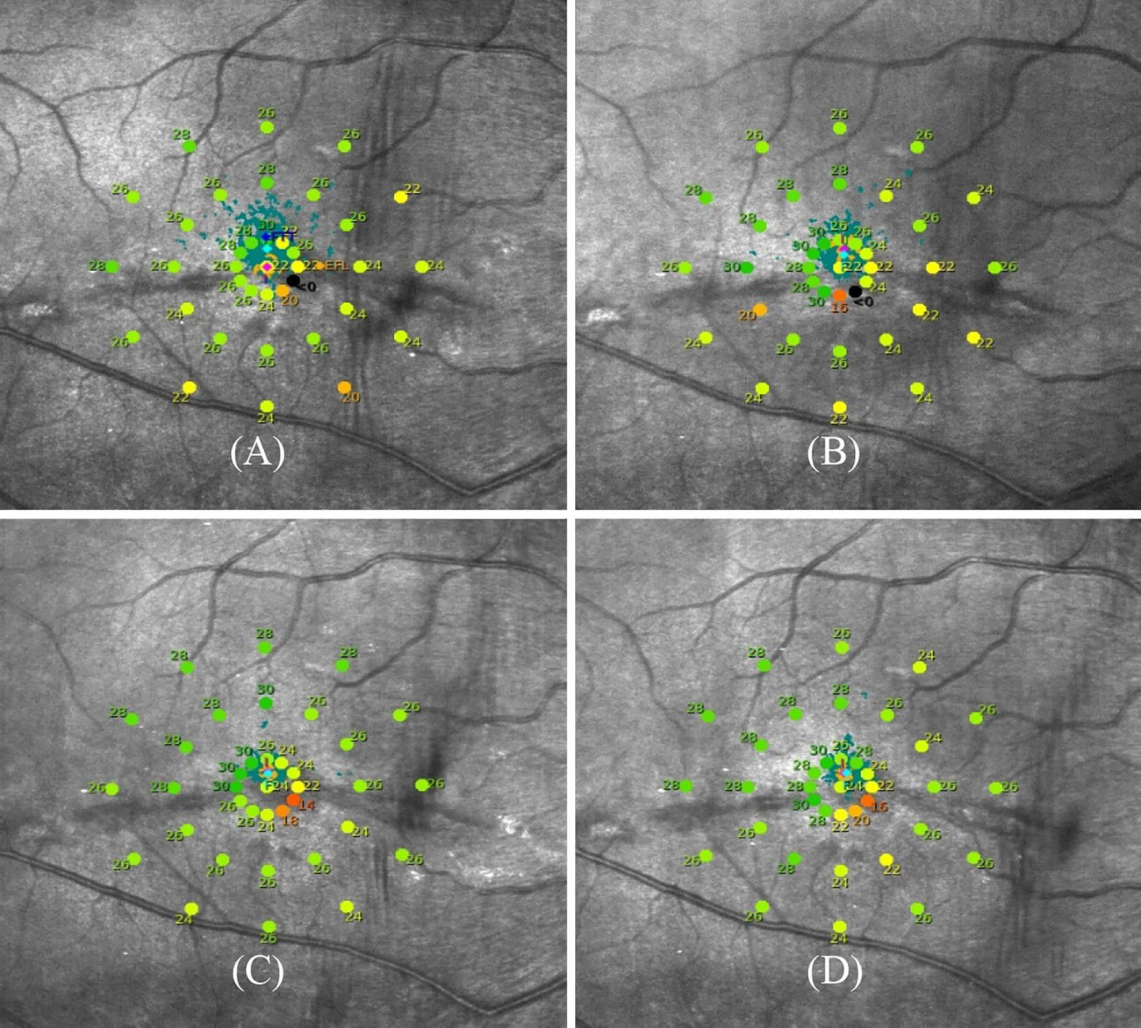
The microperimetry outcomes of a 37-year-old female who underwent RRD surgery of the right eye five years prior. The microperimetry results are shown at four different timepoints: before training (**A**), and after 5 (**B**), 10 (**C**), and 15 training sessions (D). Before training, the fixation spots were scattered (orange and light blue dots), iPRL (pink spot) and some fixation spots were located in the lesion area, and < 0 dB dark spots (black spots) were seen in 1°circle. P1 = 88%,95% BCEA Area = 4.6°^2^. BCVA = 62 letters, reading speed = 164 word/min. The 30 dB locus at 1°above the iPRL was selected as FTT (dark blue spot in figureA); after 5 training sessions, the fixation points and PRL shifted upward. P1 = 94%,95% BCEA Area = 2.6°^2^, BCVA = 66 letters, reading speed = 170 word/min. After ten training sessions, it was observed that the fixation point became more concentrated, and the 1° circle scotoma disappeared. P1 = 100%, 95% BCEA Area = 0.5°^2^, BCVA = 74 letters, reading speed = 204word/min. After fifteen training sessions, the fixation stability was slightly lower than before, but it remained concentrated. P1 = 100%,95% BCEA Area = 1.0°^2^, BCVA = 73letters, reading speed = 212 word/min.

Changes in BCVA, reading speed, P1 and LgBCEA over the training period are presented in Fig. 1 and Table 4. The BCVA and reading speed increased gradually with each training session in both groups. In group A, BCVA significantly increased after 5 sessions (P = 0.016), and continued to increase after 10 sessions(P < 0.001). Reading speed increased significantly after 15 sessions (P = 0.020). BCVA and reading speed increased significantly after 5 sessions in group B (P = 0.022, and P = 0.047, respectively), and only reading speed increased continuously after 10 sessions (P = 0.030), and then remained stable (P = 0.671). Statistically significant improvement in P1 and LgBCEA was achieved after 10 training sessions for group A (P = 0.017, and P = 0.019, respectively) and 5 training sessions for group B (P = 0.012, and P = 0.009, respectively), with no significant improvements at the next timepoint (P > 0.05). Interestingly, the change in LgBCEA after 15 sessions from baseline was significantly less in group A (− 0.18 ± 0.20) compared to Group B (− 0.37 ± 0.34;) (P = 0.047). Figure 2 illustrates the microperimetry outcomes of a 37-year-old female who underwent RRD surgery five years ago in the right eye.

**Table 4:** BCVA, reading speed and FS changes during the different training period.

Parameters	Group	Baseline	After 5 sessions	After 10 sessions	After 15 sessions	F	p
BCVA	A	31.15 ± 11.35	32.20 ± 11.40^a^	34.05 ± 12.15^ab^	34.55 ± 12.32^ab^	38.384	< 0.001
	B	36.40 ± 14.01	39.00 ± 14.09^a^	40.07 ± 15.48^a^	40.47 ± 14.86^a^	18.667	< 0.001
Reading speed	A	70.40 ± 49.59	74.00 ± 56.42	77.95 ± 60.11	84.20 ± 66.21^a^	10.715	0.002
	B	78.60 ± 57.43	82.93 ± 60.17^a^	92.60 ± 67.07^ab^	95.33 ± 69.57^ab^	15.738	< 0.001
P1	A	34.60 ± 17.25	44.40 ± 18.67	47.80 ± 16.50^a^	45.70 ± 15.81^a^	8.802	0.002
	B	60.60 ± 28.64	76.87 ± 22.17^a^	71.47 ± 23.81^a^	76.67 ± 21.54^a^	10.612	0.001
LgBCEA	A	1.39 ± 0.23	1.26 ± 0.26	1.22 ± 0.23^a^	1.21 ± 0.22^a^	9.048	0.001
	B	1.00 ± 0. 60	0.66 ± 0.45^a^	0.70 ± 0.48^a^	0.63 ± 0.52^a^	11.285	< 0.001

The superscript a indicates a statistically significant difference compared to baseline, b means statistically significant compared to 5 sessions.

## Discussion

The current study demonstrated that both daily and alternating MBFT significantly improved visual performance in patients with visual impairment, which is in line with previous findings^14,25^. However, MBFT performed on alternate days, required fewer sessions than daily training achieve the same effects. In addition, we observed different patterns of change in visual performance, and identified the optimal number of treatment sessions between the two training frequencies.

Patients with macular disease suffer from loss of central vision, scotoma in the visual field, distorted or blurred vision with reduced contrast sensitivity, and color perception. The prevalence of macular disease is rising globally, placing a significant burden on society. Preserving residual vision and enhancing patients' quality of life remain major challenge. There are currently no effective treatment options for advanced maculopathy. Although several novel treatments, including complement inhibition, neuroprotection, visual cycle modulators, stem cell-based therapies, and anti-inflammatory agents, have recently been developed, it will take time to translate these findings into clinical practice^26–28^. Currently, eccentric training is the best choice for most patients.

The MBFT has recently been applied to improve visual performance in patients with several diseases, including AMD, MMD, STGD, cone dystrophy, vitelliform dystrophy, posttraumatic macular scars, and optic neural dysfunction^14,15,25,29–32^. However, the training process is largely empirical and lacks standardization, resulting in significant variability in the recommended frequencies and number of sessions^18^. These frequencies ranged from daily to once a week, with the number of sessions ranging from 3 to 16, and the entire treatment duration ranging from 1 week to 4 months. This significant uncertainty hinders the development of a well-tailored training plan, and makes it difficult to convince patients to adhere to treatment. As such, it is essential to determine the optimal frequency and number of sessions required for this training method to establish a uniform and applicable methodology that can be adopted universally.

The primary endpoint of this study was fixation stability. We did observe both similarities and differences between the two groups. Both groups showed a small recession after reaching peak fixation stability; however, Group B showed better performance. Specifically, five sessions in Group B, consistent with the amount of days of 10 sessions in Group A, resulted in similar effects. Besides, Group B achieved a notably higher improvement in fixation stability than Group A after 15 sessions. This can be explained by the spacing effect. The spacing effect means that repetitions spaced in time tend to produce stronger memories than repetitions massed closer together, which was first proposed by Hermann Ebbinhaus^33^. The spacing effect is not only effective in language and verbal tasks but also applicable in skill-related tasks. A study investigated the effect of training frequency on suturing practice among medical students, revealing that there was a significant advantage for regular training groups (daily training, 1 session on alternative days, and weekly training) over massed training groups (3 sessions daily, and bidaily sessions)^34^. Reconsolidation appears to be involved in the mechanism underlying the spacing effect. On the one hand, the neural consolidation processes which influence the development of the long-term memory take time to develop, and the memory gets additional consolidation (reconsolidation) from the reactivation. On the other hand, the long interval of time allow more sleep, which also plays an active role in memory consolidation^35^. This suggests that we can reduce the number of times patients commute to and from the hospital for training by improving the training frequency. However, excessive spacing resulted in decreased retention^36^. In a previous study, Sahli et al.^14^ summed up their clinical experience, stating that fixation stability and patient completion rates decreased when the time between two sessions was extended. Further research is required to determine the interval that can benefit the most.

In our study, we measured the reading speed monocularly and found that it was improved for all patients, excluding three who could not be measured owning to poor near vision. Previous studies have found an improvement in reading abilities, including decreased minimum print size, greater reading speed, and reading acuity^32,37^. Unfortunately, we were unable to include other sensitive reading indicators besides reading speed in our study, as there is no Chinese version of the commonly used international reading text to measure other reading performances. Nevertheless, a previous study^38^ in patients with geographic atrophy applied linear mixed-effects models and found that reading speed reflects retinal function beyond BCVA, making it a more suitable indicator to acquire less correlated data than reading acuity. Moreover, there was a linear correlation between the better eye and binocular reading, which highlights the importance of separately testing monocular reading performance. While the fixation stability of patients did not show significant improvement at the next time point after 10 sessions in Group A or 5 sessions in Group B, there were statistically significant improvements in BCVA and reading speed. These findings suggest that continued training may play a consolidating and strengthening role in the rehabilitation of patients with macular damage. Since significant improvement was not obtained in subsequent sessions, we believe 10 sessions to be the optimal number of treatment sessions for patients undergoing alternate training.

The NEI-VFQ25 scores showed significant improvements in both groups. The most significant improvement was found in the near vision index, which is also consistent with the significant improvement in reading speed, as well as findings reported by Sahli et al.^16^.Although we did not observe a significant improvement in mental indicators in our study, we did observe that most patients were satisfied with the treatment and maintained a positive outlook. This is also a significant outcome of treatment.

The optimal location of the FTT has been previously investigated. In one study, Morales et al.^10^ compared the outcomes of PRL and TRL, and found that VA, FS, and reading speed significantly improved only in the TRL group. However, the authors did not make personalized choices based on the patient's condition. Our previous study found that natural PRL may be optimal for patients with small and sharp scotomas, whereas patients with large and rough scotomas need to develop new training target^24^. In our study, four patients in whom the original PRL was used also improved visual performance, providing additional validation for our prior assertion that training targets should be selected individually.

Another puzzling problem is the choice of training eyes. The optimal eye for the MBFT is unclear in patients with inconsistent biocular macular disease. Vision specialists have typically opted to perform rehabilitation in one eye, leaving open the questions of binocular behavior and rehabilitation. Morales et al.^39^ explained why the MBFT has mostly been performed monocularly, stating that as microperimetry examination and training are performed in one eye at a time, and abnormal retinal diplopia can be caused by the asymmetry of the PRL position in both eyes. Previous studies have suggested that the fixation and PRL location of the worse eye (WE) depend on the better eye (BE)^40,41^, leading most studies to perform MBFT on the BE. In contrast, Oflaz et al.^16^ chose WE for training because it consumed better reorganization capacity in worse eyes. In our study, we performed the MBFT in both eyes with biocular inclusion in 6 patients. In these patients, WE and BE improved equally with fixation, and none of them complained of diplopia. Our experience has proven that binocular training can benefit both eyes.

Several studies have previously provided evidence that MBFT can result in physiological or structural changes in patients with macular diseases. For example, Vingolo et al.^42^ demonstrated that the visual-evoked potential(VEP) P100 amplitude increased in patients with myopic maculopathy after 10 training sessions with MP-1 biofeedback (7 min) and Visual Pathfinder (3 min). They further suggested that the MBFT could re-reference the oculomotor system to the functional retinal locus and induce cortical reorganization in adult patients. Furthermore, a randomized controlled trial carried out by Melillo^37^, who compared a group treated with the MBFT to a control group of 12 patients with STGD, showed that after 12 weekly treatment sessions, fMRI showed a significant effect (P < 0.001) of biofeedback on primary visual cortex activation in the treated group compared to the control group, providing further support for the phenomenon of neuronal reorganization induced by MBFT. It is worth noting that MBFT is a rehabilitation method for improving residual vision without utilizing low-vision devices. These studies underscore the potential of biofeedback training as a promising technique for enhancing the visual function in patients with macular diseases. Unfortunately, when the change occurred and how to make that change greater were not known.

To the best of our knowledge, this is the first study to compare outcome at different time points in patients treated with MBFT. The rigorous and intensive MBFT regimen coupled with sufficient treatment sessions enabled us to isolate the effects of the therapy from the natural progression of the underlying condition. These findings highlight the importance of distinguishing frequencies when designing MBFT plans for patients. Our research thus provides a protocol that can be applied to clinical and scientific studies, focusing on the overall response at different training times and frequencies to further guide personalized treatment.

Nevertheless, the study has several important limitations that need to be considered. First, we failed to perform random grouping because the training required patients to travel to and from the hospital many times, which depends on patient time coordination, resulting in the fixation ability at baseline differing considerably between the groups. Given the inconsistent baseline levels of fixation stability, we compared the change in values from the baseline of the two groups to mitigate potential confounding factors and to determine which group had a better treatment effect. Second, our sample size was relatively small, and recruiting patients during the novel coronavirus epidemic is particularly challenging; the small number of enrolled patients did not enable us to perform a statistical analysis stratified by age, disease type, and lesion size, nor to compare the rest of the training frequency. In addition, we did not conduct a long-term follow-up and were unable to prove the long-term effects. Despite these limitations, the study results are expected to form the basis for larger-scale controlled clinical trials in the future.

In conclusion, we provided further evidence that the MBFT is a practical, repeatable, and non-invasive method that improves residual visual function in patients with macular diseases. Besides, we provide a complete MBFT program. Regarding the effect of training frequency, the improvement in fixation stability was more evident in the extended interval group, but the BCVA, reading speed, and NEI-VFQ 25 scores showed similar benefits in both groups. Given that longer treatment time burdens patients, properly prolonging the training interval could maximize patients benefits. Achieving the best effect requires approximately 15 sessions for daily training and 10 sessions for alternate daily training. Simultaneous binocular MBFT can provide binocular benefits to patients with binocular lesions. MBFT is a promising treatment for patients with macular disease who currently have no effective treatment; however, its exact mechanism and best scheme still need to be further explored and studied. More high-quality, large-samples, long-term follow-up, randomized controlled trials are needed to determine the most effective treatment strategy.

### Supplementary Information

Supplementary Information.

## Data Availability

The data supporting the findings of this study are available from the corresponding author upon reasonable request.
