# Supplementary material for: The effect of duration between sessions on microperimetric biofeedback training in patients with maculopathies
Source: Sci Rep. 2024 May 31;14:12524. doi: 10.1038/s41598-024-63327-x (PMC11143284; doi:10.1038/s41598-024-63327-x)
Supplement: Supplementary file 1 — Supplementary Information. [file 41598_2024_63327_MOESM1_ESM.docx]

**Materials and methods**

In this prospective non-randomized controlled study, all participants met the inclusion and exclusion criteria. Prior to training, all subjects underwent relevant ocular examinations, including uncorrected visual acuity, best corrected visual acuity (measured using a standard logarithmic visual acuity chart), non-contact tonometry (Topcon CT-1, Japan), computerized refraction (KR-800, TOPCON, Japan), slit lamp examination of the anterior segment, fundus examination with a non-contact lens (VOLK 90D, USA), ultra-widefield scanning laser ophthalmoscopy (Daytona, Optos, UK), and OCT (Spectralis HRA+OCT, Heidelberg, Germany), among other baseline assessments. The key outcome measures are as follows:

1. BCVA

The best corrected visual acuity (BCVA) was measured using the Early Treatment Diabetic Retinopathy Study chart (ETDRS) standard visual acuity chart (ESV3000, Good-Lite, USA). The measurement method involved the subject sitting in a chair at a distance of 4 meters, ensuring that the height of the tested eye was aligned with the 0.0 logMAR line on the visual acuity chart and occluding the opposite eye. If both eyes underwent training, the eye with better vision was measured first, followed by the eye with poorer vision. After optimal refractive correction, starting with the largest letter, the subject identified the letters line by line. If ≥20 letters were correctly identified at 4 meters, 30 additional letters were added to the total number of letters; if <20 letters were correctly identified at 4 meters, the subject moved to 1 meter, and all correctly identified letters were recorded. The total number of correctly identified letters was calculated as the sum of letters correctly identified at 4 meters and those identified at 1 meter.


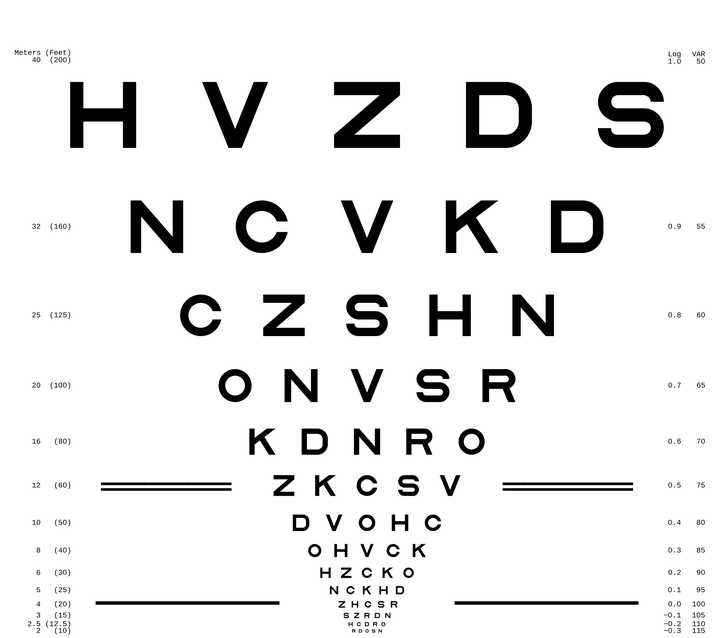


Figure1. the Early Treatment Diabetic Retinopathy Study chart

1. Reading speed

In this study, the Chinese version of the International Reading Speed Texts (IReST), provided by Hartest Precision Instruments, UK, was used for measuring reading speed.The reading speed measurement method involved patients undergoing optical correction based on their best corrected near vision in the same room, position, and lighting conditions. Patients held the IReST reading card with both hands, read at a distance of 40 cm, and were briefed on the reading rules using the second passage as an example. Random passages from the IReST were selected, timing commenced upon revealing the passage, errors were not corrected, and any mistakes or omissions were noted. Reading time was recorded using a stopwatch, with each measurement recording the passage number, time spent reading (in seconds), number of correctly read words, and calculated reading speed (words/min). Follow-up assessments utilized passages matched for difficulty under the same conditions.


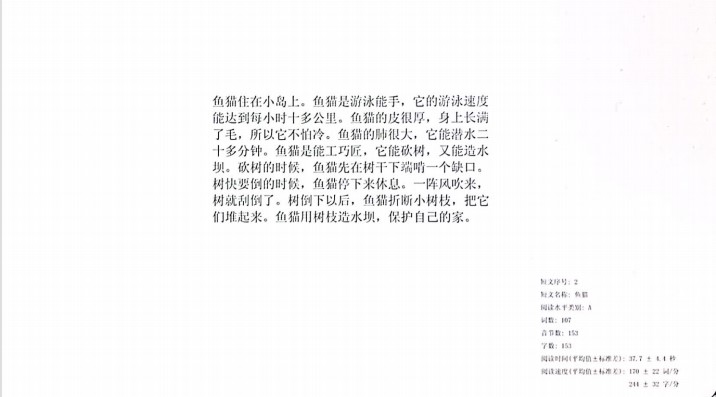


Figure2. Chinese version of International Reading Speed Texts

1. Microperimetry

This study utilized the MAIA microperimeter (CenterVue, Padova, Italy) for microperimetry examinations. The MAIA microperimeter employs a linear scanning laser ophthalmoscopy (SLO) mode to provide non-mydriatic and high-quality real-time retinal images. Through real-time image calibration and a 25 frames per second eye-tracking system, it automatically compensates for eye movements, accurately correlating retinal structure with visual function. The device directly measures and displays precise light sensitivity, fixation stability and location, preferred retinal locus (PRL), as well as the specific location and size of scotomas on the retina. The working distance of the MAIA microperimeter is 30 mm, with a background luminance of 4 asb, a red circular fixation target, Goldmann III stimulus size, stimulus luminance ranging from 0.25 asb to 1000 asb, stimulus duration of 200 ms, and a stimulus dynamic range of 0-36 dB. The examination protocol selected expert mode with full threshold 4-2 testing strategy, a measurement range of 10°, 37 stimulus points including one at the initial PRL (iPRL) within the first 10 seconds, and concentric circles with radii of 1°, 3°, and 5° centered around the iPRL, each containing 12 stimulus points. Patient dilation was not required for the examination, and patients with refractive errors exceeding the automatic focusing range of the machine (-15 D to +10 D) wore corrective lenses for testing under best corrected visual acuity. Subjects adapted to the dark room for 10 minutes before testing. An occluder provided by the instrument was used to shield the other eye during examination, and if bilateral examination was necessary, the eye with better vision was tested first, followed by the other eye if both eyes had consistent visual acuity. The following parameters were recorded: proportion of fixation points within a 2° diameter (P1), proportion of fixation points within a 4° diameter (P2), 63% bivariate contour ellipse area (BCEA) (63%BCEA), and 95% BCEA (95%BCEA) of fixation points (as shown in Figure 3-1). Follow-up examinations were conducted using the follow-up mode.


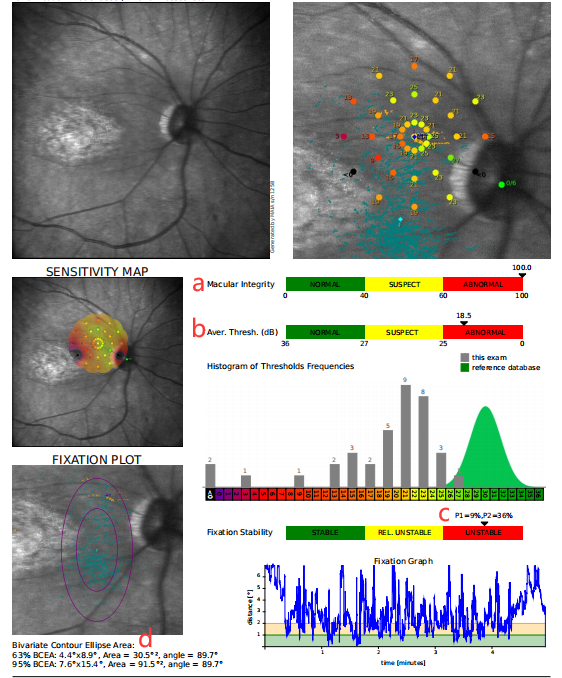


Figure 3-1. Microperimetry examination results

a. Macular integrity; b. Mean retinal threshold; c. Fixation stability indices P1 and P2; d. Fixation stability indices 63%BCEA and 95%BCEA; Blue dots indicated by orange arrows: PRL (Preferred Retinal Locus).

1. Visual Function Questionnaire

The NEI VFQ-25 consists of 12 domains and 25 questions related to visual function, including general health status (1 item), overall vision (1 item), eye pain (2 items), near activities (3 items), distance activities (3 items), peripheral vision (1 item), color vision (1 item), driving (2 items), social functioning (2 items), role limitations due to vision problems (2 items), dependency (3 items), and mental health (4 items). When patients have reduced vision, the questionnaire is administered orally by the same ophthalmologist, who records the patient's responses accordingly. Otherwise, patients complete the questionnaire independently. Responses are scored on a 5-point scale based on the patient's subjective perception, ranging from 0 to 4, representing the percentage of the task the patient can complete: 0% (unable to complete due to vision impairment), 25% (very difficult), 50% (moderately difficult), 75% (slightly difficult), and 100% (no difficulty). Finally, the scores of each item are summed to reflect the patient's quality of life, with higher scores indicating better quality of life.

5.**Procedure.** The MBFT training process consists of two parts:

5.1.Choose the Fixation Training Target(FTT): The selection of FTT is according to the microperimetry result and based on the following criteria: (1) PRL as FTT if the natural PRL is within an area of good retinal sensitivity and in a convenient location or (2) set a FTT with good retinal sensitivity, closest to the fovea, and choose the up side of natural PRL as far as possible, which present the inferior visual field; This criteria is based on our extensive literature review and clinical experience spanning several years.

5.2. Perform MBFT: The built-in MBFT module of MAIA microperimetry was utilized for training. During training, patients were instructed to rotate their eyeballs to find the 1-degree area of FTT and maintained as long as possible. Once they fixed at the fixation target, the sound of machine turned to a continuous beep with a white light on, and these feedback help patients maintained a high level of attention. The experienced trainers supervised the training process, which consisted of 15 sessions, each lasting 10 minutes. The training sessions were conducted on every day or on every other day based on patients’ time availability to commute to hospital and receive training.


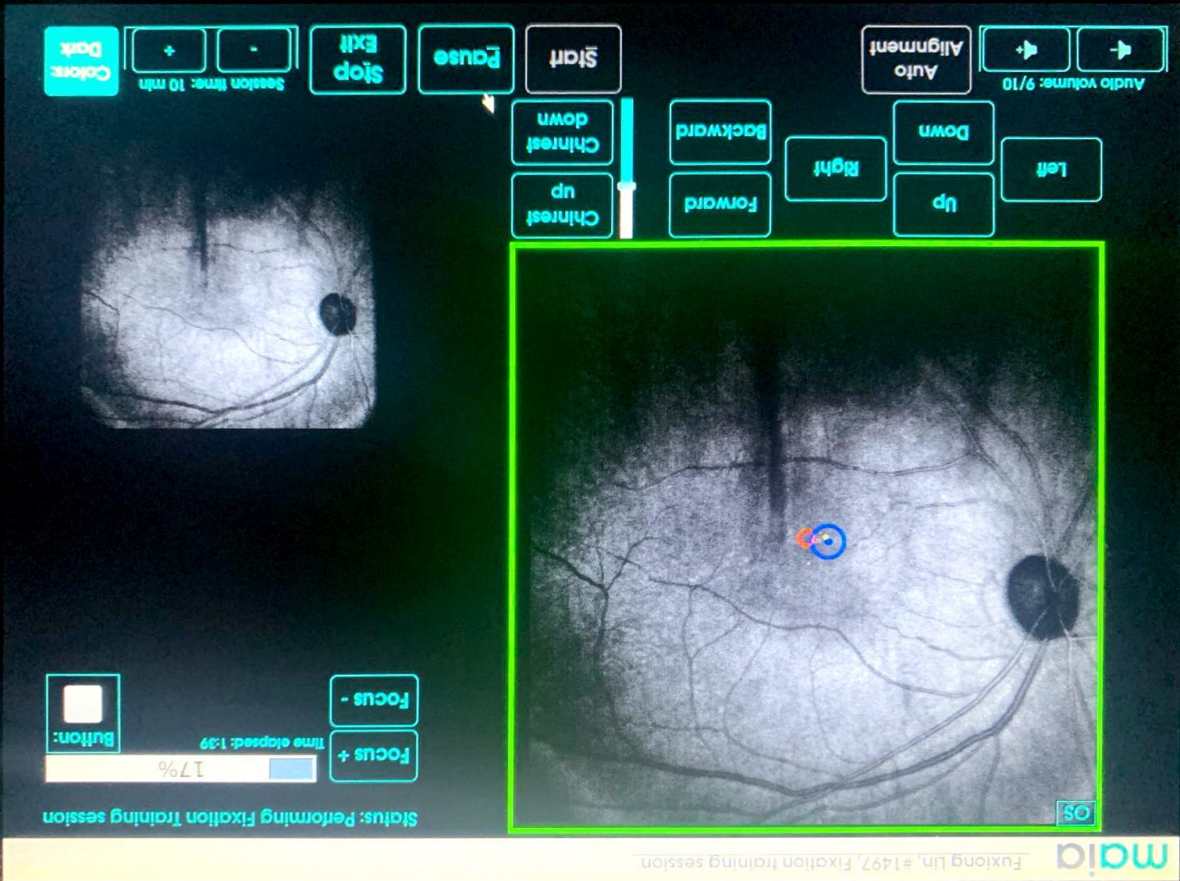


Figure4. Microperimetry biofeedback training

The blue circle denoted by the blue arrow represents the fixation target location (FTT) of the patient within a 1° radius centered on the blue circle. The red circle indicated by the red arrow represents the stimulus laser spot. The examiner is required to instruct the patient to maintain fixation on the designated point (yellow dot) within the blue circle.
